# Chinese herbal medicine combination therapy for patients with steroid-dependent ulcerative colitis: Study protocol for a systematic review and meta-analysis

**DOI:** 10.1097/MD.0000000000019729

**Published:** 2020-04-17

**Authors:** Qiaobo Ye, Zhipeng Hu, Maoyi Yang, Kaihua Qin, Yingguang Zhou

**Affiliations:** a Basic Medical College, Chengdu University of Traditional Chinese Medicine; b Hospital of Chengdu University of Traditional Chinese Medicine; c Health Preservation and Rehabilitation College, Chengdu University of Traditional Chinese Medicine, Chengdu, China.

**Keywords:** Chinese herbal medicine, meta-analysis, protocol, systematic review, Ulcerative Colitis

## Abstract

**Background:**

Ulcerative colitis (UC) is a chronic non-specific intestinal inflammatory disease characterized by continuous and diffuse inflammatory response of colonic mucosa. Steroid-dependent UC is an important type of UC. Chinese herbal medicine is widely used in treating steroid-dependent UC in China. However, there is no systematic review and meta-analysis to collate and evaluate the evidence of these studies. The purpose of this research is to provide evidence of the efficacy and safety of Chinese herbal medicine in treating steroid-dependent UC.

**Methods and analysis:**

Six databases, including 3 English databases and 3 Chinese databases will be searched. In addition, other grey literatures and ongoing studies will also be searched. Two researchers will independently select eligible studies by reading titles, abstracts and full texts according to the inclusion and exclusion criteria. Risk of bias assessment will be conducted by 2 independent reviewers using Cochrane risk-of-bias tool. The outcomes include steroid-free remission rate, Total clinical effective rate, Incidence of adverse events, Disease activity index (modified Mayo score), Results of enteroscopy (Baron score) and mucosa (geboes index score). Heterogeneity between studies will be assessed by Cochrane *X*^2^ and *I*^*2*^ tests. We will conduct subgroup analysis and meta-regression to explore the source of heterogeneity. We will also evaluate the stability of the results through sensitivity analysis and publication bias through funnel plot and Egger test.

**Results:**

The results will be published in peer-reviewed journals.

**Conclusion:**

Our meta-analysis and systematic evaluation results will confirm whether Chinese herbal medicine is effective in the treatment of steroid-dependent UC. It will provide more ideas for future research.

**OSF registration number:**

DOI: 10.17605/OSF.IO/YP79Z

## 1 Introduction

Ulcerative colitis (UC) is a chronic non-specific inflammation disease characterized by abdominal pain and diarrhea.^[1–3]^ Overall, the incidence of UC is higher in developed China than that in developing countries. The prevalence of UC in north America and Europe is 24.30/10000 and 19.20/10000 respectively. The prevalence of UC in Asia and the Middle East is lower, about 6.30/10000. The prevalence of UC in mainland of China is about 11.6/10000.^[4]^ Many complications occur in the later stage of UC, including toxic megacolon, intestinal perforation, lower gastrointestinal hemorrhage, intraepithelial neoplasia and colorectal cancer.^[5,6]^ The recurrence of UC brings a heavy economic and health burden to the health system and patients.

At present, the clinical treatment of UC includes 5-ASA (such as sulfasalazine, sulfasalazine, and so on), glucocorticoids (such as prednisone, hydrocortisone, and so on), immunosuppressants (azathioprine, cyclosporine, and so on), biological agents and surgical treatment.^[7–10]^ Among these medicines, steroid is an effective treatment for UC, especially for moderate to severely UC. However, steroids therapy has some defects. First, steroids therapy can only be used to induce remission, but not as a maintain therapy. Second, the condition is easy to relapse after the cessation of treatment, and the patient eventually becomes steroid-dependent UC.^[11]^ In addition, there are many adverse effects in the long-term use of steroid. Therefore, how to treat the patients with steroid-dependence has become a clinical difficulty.

Chinese herbal medicine (CHM) has a long history in treating UC.^[12]^ In China, many steroids-dependent UC patients receive CHM for adjuvant treatment, hoping to reduce the dosage of steroid and adverse effects. Many clinical studies about the effect of CHM for steroids-dependent UC have been carried out. In these studies, traditional Chinese medicine can improve the condition of patients.^[13–18]^ However, due to the small sample size and the defects of study design, these clinical studies have failed to give a comprehensive and definite answer to the application of CHM in steroid-dependent UC.

In this study, we will systematically summarize the clinical evidence of CHM in treating steroid-dependent UC. For clinicians, this study will provide evidence-based guidance for the clinical application of CHM on steroid-dependent UC. For researchers, this study will point out the existing gaps in the current research of CHM in the treatment of steroid-dependent UC, and provide direction for the future research.

## 2 Methods and analysis

### 2.1 Study registration

This study has been registered on Open Science Framework (OSF) and the registration number is DOI: 10.17605/OSF.IO/YP79Z (registration information is available at https://osf.io/yp79z). This study protocol is reported according to the guidelines recommended in the Preferred Reporting Items for Systematic Reviews and Meta-analysis Protocols checklist.^[19]^

### 2.2 Inclusion criteria

#### 2.2.1 Study design

We will only include randomized controlled trials (RCTs). Non-RCT studies, for example, cohort studies and retrospective studies will not be included in our research. Similarly, real world studies and cluster RCTs will not be included. In addition, conference, abstract and review articles will not be included due to the lack of detailed information.

#### 2.2.2 Participants

Participants with an established diagnose of steroid-dependent UC will be included in our study. There will be no restriction about age, region, and gender of participants.

#### 2.2.3 Types of intervention

All type of CHM, whether single herbal medicine or formula, will be included. There will be no restriction about treatment course, frequency and dosage.

#### 2.2.4 Types of controls

The control group can use blank control, placebo control, conventional treatment control, or no treatment. In addition to CHM, patients in the experimental group and the control group must receive the same treatment.

#### 2.2.5 Outcomes

##### 2.2.5.1 Main outcome

Since the participants of this study are patients with steroid-dependent UC, the main outcome of this study is the steroid free remission rate of the 2 groups. There will be no time limitation about main outcome.

##### 2.2.5.2 Secondary outcomes

(1) Total clinical effective rate.
(2) Incidence of adverse events.
(3) Disease activity index (modified Mayo score).
(4) Results of enteroscopy (Baron score).
(5) Mucosa (geboes index score).

Scale score, laboratory test and enteroscopy will be performed before and after treatment. There will be no limit about the measurement of results.

### 2.3 Study search

Three English databases including PubMed, EMBASE and Cochrane Library and 3 Chinese databases including China National Knowledge Infrastructure, Wanfang Data knowledge service platform and VIP information resource integration service platform will be searched from its inception to March 2020 without language limitation. In addition, we will search Google Scholar and Baidu scholar for more related researches. And above all, the Chinese Clinical Trial Registration Center and clinical trials.gov will be searched to find out more ongoing studies. The combination of MeSH terms and free-text words will be adopted by us for database searching.

We will mainly use the following terms to search: (“chinese herbal medicine” OR “Chinese Herbal Medicine” OR “Drugs, Chinese Herbal” OR “Chinese Herbal” OR “Chinese herbal” OR “Medicine, Chinese Traditional” OR “Traditional Chinese Medicine” OR “traditional chinese medicine” OR “Traditional Chinese medicine decoction”) AND (“Ulcerative Colitis” OR“Colitis, Ulcerative” OR “Idiopathic Proctocolitis” OR“Colitis Gravis”OR“Inflammatory Bowel Disease, Ulcerative Colitis Type”). The work will be independently conducted by 2 authors (Qiaobo Ye and Maoyi Yang). The search process of the PubMed is presented in Table 1.

**Table 1:**
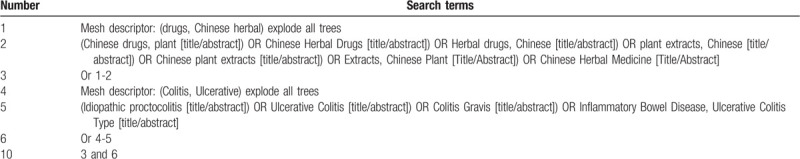
Example of PubMed search strategy.

### 2.4 Study selection

**Figure 1:**
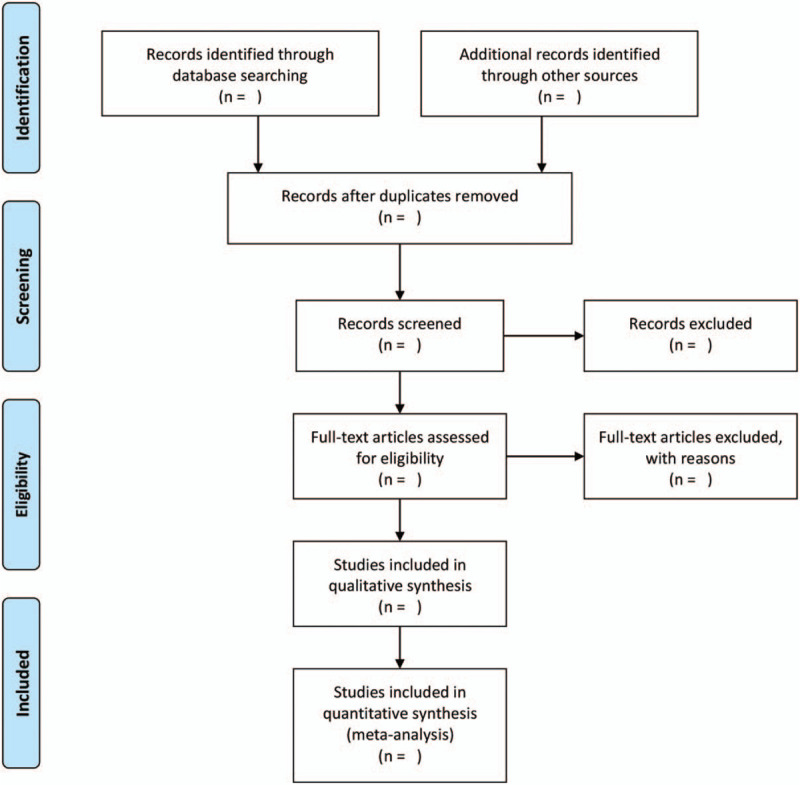
Flow chart of study selection.

We will use EndNote X8 for Mac to manage the citations. Two researchers (Kaihua Qin and Yingguang Zhou) will screen citations independently. Disagreement between 2 authors will be solved by discussion with a third author (Zhipeng Hu). A research flow chart will be drawn to show the whole process of research selection (Fig. 1).

### 2.5 Data extraction

Data in studies will be extracted into Microsoft Excel by 2 independent researchers. We will extract the following data: the first author's name, publication time, article title, interventions in experimental and control group, course of treatment, course of disease, number of patients in each group, ages, and sex of patients, outcomes and adverse effect. If some information is not clearly described in studies, we will contact the authors for further explanation.

### 2.6 Risk of bias assessment

The risk of bias will be assessed by Cochrane risk-of-bias tool. This is a domain-based tool which is widely used in both Cochrane reviews and non-Cochrane reviews. In this tool, there are 7 domains: random sequence generation (selection bias), allocation concealment (selection bias), blinding of participants and personnel (performance bias), blinding of outcome assessment (detection bias), incomplete outcome data (attrition bias), selective reporting (reporting bias), other bias. A completely transparent judgement will be made by 2 authors (Qiaobo Ye and Kaihua Qin). Any disagreement will be solved by consensus with a third author (Zhipeng Hu).

### 2.7 Data analysis

Data analysis will be conducted using Review Manager Version 5.3 and Stata 14.0 software. The effect measure of binary variable will be expressed as risk ratio and 95% confidence interval, and calculated by mantel Haenszel method. For continuous variables, 95% confidence interval and mean difference or standardized mean difference (SMD) will be used. We will use Cochrane *X*^2^ and *I*^*2*^ tests^[20]^ to complete the heterogeneity analysis of the study. When *P* < .05 and *I*^*2*^ > 50%, we will use the random effect model. When *P* > .05 and *I*^*2*^ < 50%, the heterogeneity can be ignored, indicating the included studies are homogeneous and the differences between them can be ignored, we will use fixed effect model to calculate the effect size. If there is a substantial heterogeneity and quantitative synthesis is not appropriate, the results will be presented in the form of tables and figures.

### 2.8 Investigation of heterogeneity

If there is substantial heterogeneity between studies, then we will conduct subgroup analysis to explore the source of heterogeneity.^[21]^ Subgroup analysis will be performed based on the following hypotheses: baseline condition, duration of intervention, type of control^.^^[22,23]^ Meta-regression will be performed if enough studies are included.

### 2.9 Sensitivity analysis

We will evaluate the stability of the results by sensitivity analysis. We will exclude each research included in the analysis 1 by 1, and then compare the results with the original research results to observe the differences between the 2 results. Using this method, we can evaluate the impact of each study on the overall research results, and then determine the reliability of the results.

### 2.10 Publication bias assessment

If more than 10 studies are included, we will assess publication bias by funnel plot. If funnel plot is symmetrical, then there is no publication bias. If funnel plot is asymmetric, it means there is a publication bias. We will also conduct the Egger test to further statistically assess publication bias. If *P* < .05, then there is publication bias.^[24]^

### 2.11 Ethics and dissemination

Meta-analysis is an analysis of previous research data and does not require ethical approval. The results of this study will be published in peer-reviewed journals.

## 3 Discussion

In recent years, due to the improvement of living standard and the change of lifestyle, the incidence of UC has increased dramatically. Steroids are widely used in the induction therapy of UC. With the increasing use of steroids, more and more patients become steroid-dependent. CHM has a long history and rich experience for UC.^[25]^ In China, more and more steroid-dependent UC patients seek for CHM treatment. However, there is no meta-analysis to systematically collect and evaluate these research evidence. In order to provide strong evidence-based medicine support for the future research and clinical application of CHM in the treatment of steroid-dependent UC, we will this systematic review and meta-analysis.

In this study, in order to collect evidence more comprehensively, there will be not restriction about the type of Chinese herbs. The Cochrane risk-of-bias tool will be used to assess the risk of bias of the included studies. In addition, we will conduct subgroup analysis to further explore the source of heterogeneity and the possible factors that can affect the effect of treatment. By doing this, we will able to provide precise empirical evidence for clinical trial and applications for CHM.

### 3.1 Amendments

If any modification is required, we will update our protocol to include any changes in the entire research process.

## Author contributions

The scheme was designed by QY, MY, and ZH. All the authors participated in the study. All authors approved the final manuscript before the article was submitted. QY, MY, and ZH contribute the same to this work and should be regarded as the co first author.

**Conceptualization:** Qiaobo Ye, Maoyi Yang, Zhipeng Hu.

**Data curation:** Qiaobo Ye, Maoyi Yang.

**Formal analysis:** Maoyi Yang, Zhipeng Hu.

**Investigation:** Qiaobo Ye, Kaihua Qin, Yingguang Zhou.

**Methodology:** Qiaobo Ye, Maoyi Yang, Zhipeng Hu.

**Project administration:** Zhipeng Hu.

**Software:** Qiaobo Ye, Kaihua Qin.

**Visualization:** Kaihua Qin.

**Writing – original draft:** Maoyi Yang.

**Writing – review & editing:** Qiaobo Ye and Yingguang Zhou.

## References

[1] Freeman HJ Pouchitis-associated iritis (uveitis) following total proctocolectomy and ileal pouch-to-anal anastomosis in ulcerative colitis. Can J Gastroenterol 2016;15:131–3. 10.1155/2001/691746 11240384

[2] Steinhart AH Fernandes A Clinical practice guidelines for the medical management of nonhospitalized ulcerative colitis:the patient perspective. Can J Gastroenterol Hepatol 2016;29:294–6. 10.1155/2015/214937 PMC4578450 26000730

[3] Risques RA Lai LA Himmetoglu C Ulcerative colitis-associated colorectal cancer arises in a field of short telomeres, senescence, and inflammation. Cancer Res 2015;71:1669–79. 10.1158/0008-5472.CAN-10-1966 PMC3077943 21363920

[4] Ng SC Shi HY Hamidi N Worldwide incidence and prevalence of inflammatory bowel disease in the 21st century: a systematic review of population-based studies. Lancet 2017;390:2769–78. 29050646 10.1016/S0140-6736(17)32448-0

[5] Zhang YZ Li YY Inflammatory bowel disease: pathogenesis. World J Gastroenterol 2014;20:91–9. 24415861 10.3748/wjg.v20.i1.91 PMC3886036

[6] Hodson R Inflammatory bowel disease. Nature 2016;540:S97. 28002398 10.1038/540S97a

[7] Gjuladin-Hellon T Gordon M Iheozor-Ejiofor Z Oral 5-aminosalicylic acid for maintenance of surgically-induced remission in Crohn's disease. Cochrane Database Syst Rev 2019;6:CD008414. 31220875 10.1002/14651858.CD008414.pub3 PMC6586553

[8] Lim WC Wang Y MacDonald JK Aminosalicylates for induction of remission or response in Crohn's disease. Cochrane Database Syst Rev 2016;7:Cd008870. 27372735 10.1002/14651858.CD008870.pub2 PMC6457996

[9] Benchimol EI Seow CH Steinhart AH Traditional corticosteroids for induction of remission in Crohn's disease. Cochrane Database Syst Rev (Online) 2008;2:CD006792. 10.1002/14651858.CD006792.pub2 PMC6718222 18425970

[10] Feagan BG Rutgeerts P Sands BE Vedolizumab as induction and maintenance therapy for ulcerative colitis. N Engl J Med 2013;369:699–710. 23964932 10.1056/NEJMoa1215734

[11] Armuzzi Alessandro Pugliese Daniela Danese Silvio Infliximab in steroid-dependent ulcerative colitis: effectiveness and predictors of clinical and endoscopic remission. Inflamm Bowel Dis 2013;19:1065–72. 23448790 10.1097/MIB.0b013e3182802909

[12] Fan Y Yi W Huang H Efficacy of herbal medicine (Gegen Qinlian Decoction) on ulcerative colitis: a systematic review of randomized controlled trials. Medicine 2019;98: 10.1097/MD.0000000000018512 PMC6946546 31876740

[13] Shandong University of traditional Chinese medicine, Xue Y Clinical research and Mechanism Discussion on the treatment of hormone dependent ulcerative colitis with jiawei’anchangyuyang decoction. 2014.

[14] Nanjing University of traditional Chinese medicine, Yang J Study on the evaluation of the therapeutic effect of Jianpi Shugan method on maintaining the syndrome of liver depression and spleen deficiency in ulcerative colitis. 2018.

[15] Beijing University of traditional Chinese medicine, Yang Z Clinical characteristics of hormone refractory ulcerative colitis and observation of therapeutic effect of integrated traditional Chinese and Western medicine. 2018.

[16] Zhang Y Guo Q Zheng l Clinical efficacy of Jianpi Qingchang formula in the treatment of ulcerative colitis with hormone dependence of spleen deficiency and damp heat type. Chinese Journal of experimental pharmacology 2019;025:69–73.

[17] Zhang Y Zheng l Guo Q Effect of Jianpi Qingchang formula on hormone withdrawal in patients with ulcerative colitis of spleen deficiency and damp heat type. Chinese Journal of experimental prescriptions 2020;26:109–13.

[18] Zheng K Shen H Wang F Clinical study on the treatment of hormone dependent ulcerative colitis by oral administration of Jianpi Bushen, Qingchang Lianyang Fang and Enema of traditional Chinese medicine. Hebei Traditional Chinese medicine 2019;41:817–21.

[19] Moher D Shamseer L Clarke M Preferred Reporting Items for Systematic Review and Meta-Analysis Protocols (PRISMA-P) 2015 statement. Syst Rev 2015;4:1. 25554246 10.1186/2046-4053-4-1 PMC4320440

[20] Higgins JPT Thompson SG fying heterogeneity in a metaanalysis. Statistics in Medicine 2002;21:15391558. 10.1002/sim.1186 12111919

[21] Sun X Briel M Walter SD Is a subgroup effect believable? Updating criteria to evaluate the credibility of subgroup analyses. BMJ 2010;340:c117. 20354011 10.1136/bmj.c117

[22] Oxman D Guyatt GH A consumer's guide to subgroup analyses. Ann Intern Med 1992;116:78–84. 1530753 10.7326/0003-4819-116-1-78

[23] Egger M Davey Smith G Schneider M Bias in meta-analysis detected by a simple, graphical test. BMJ 1997;315:629–34. 9310563 10.1136/bmj.315.7109.629 PMC2127453

[24] Peters JL Sutton AJ Jones DR Contour-enhanced meta-analysis funnel plots help distinguish publication bias from other causes of asymmetry. J Clin Epidemiol 2008;61:991–6. 18538991 10.1016/j.jclinepi.2007.11.010

[25] Ke F Yadav PK Ju Liu Z Herbal medicine in the treatment of ulcerative colitis. The Saudi Journal of Gastroenterology 2012;18:349–51. 22249085 10.4103/1319-3767.91726 PMC3271691

